# Critical windows and risk thresholds of prenatal mixed air pollutant exposure for oligohydramnios: Evidence from a population‑based study

**DOI:** 10.1097/EE9.0000000000000454

**Published:** 2026-02-12

**Authors:** Sijing Zhu, Jiayuan Feng, Hui Niu, Liu Fang, Xiaoxue Li, Yin Yang, Jinlu Liang, Ziyi Chen, Mengfei Sun, Lu Zong, Wenfang Yang, Mei Chun Chung

**Affiliations:** a Department of Obstetrics and Gynecology, The First Affiliated Hospital of Xi’an Jiaotong University, Xi’an, Shaanxi, People’s Republic of China; b School of Public Health, Xi’an Jiaotong University Health Science Center, Xi’an, Shaanxi, People’s Republic of China; c Division of Nutrition Epidemiology and Data Science, Friedman School of Nutrition Science and Policy, Tufts University, Massachusetts Boston

**Keywords:** Oligohydramnios, Ambient air pollution, Mixture exposure, Critical exposure windows, Preventive thresholds, Acute exposure effects

## Abstract

**Background:**

Oligohydramnios is a clinically relevant but understudied pregnancy complication. This study evaluated the association between maternal exposure to mixed ambient air pollutants and the risk of oligohydramnios, focusing on identifying critical exposure windows and pollutant-specific concentration thresholds.

**Methods:**

We conducted a retrospective cohort study of 7,608 singleton live births from a tertiary hospital in northwestern China (2015–2019). Individual-level air pollution exposure was estimated by inverse distance weighting. Weighted quantile sum (WQS) and lagged WQS (lWQS) models were used to assess mixture effects and time-specific susceptibility. Restricted cubic spline models were applied to estimate concentration–response relationships and preventive thresholds of representative weeks and corresponding key pollutants.

**Results:**

The WQS index showed a significant joint effect for daily average exposure during whole pregnancy (odds ratio = 1.204, 95% confidene interval 1.049, 1.285), mainly driven by NO_2_ and O_3_. The lWQS model identified the early and late pregnancy as critical exposure windows. As representative time points for early, mid, and late pregnancy, estimated O_3_ thresholds were 49.28 μg/m^3^ (week 4), 36.28 μg/m^3^ (week 16), and 37.40 μg/m^3^ (week 32); the NO_2_ threshold at week 32 was 37.41 μg/m^3^.

**Conclusion:**

Maternal exposure to mixed air pollutants, particularly O_3_ and NO_2_, increases the risk of oligohydramnios. Findings highlight gestational timing and pollutant-specific targets for prenatal environmental protection.

**What this study adds**

This study is the first to investigate the relationship between ambient air pollution mixtures and oligohydramnios risk. Using a well-characterized birth cohort, it integrates weighted quantile sum, lagged weighted quantile sum, and restricted cubic spline models to capture pollutant-specific, time-sensitive, and nonlinear exposure effects. This methodological framework enabled the identification of dominant pollutants, critical exposure windows, and preventive thresholds—providing both methodological innovation and actionable insights for prenatal environmental health interventions. Notably, health risks were detected even at pollutant levels below current air quality standards, suggesting potential gaps in protection for pregnant populations.

## Introduction

In recent years, numerous epidemiologic and mechanistic experimental evidence have suggested that maternal exposure to ambient air pollution can adversely affect pregnancy, contributing to outcomes such as gestational hypertension, preterm birth, and fetal growth restriction through pathways involving oxidative stress, placental vascular dysfunction, and systemic inflammation.^1,2^ In contrast, oligohydramnios, a reduction in amniotic fluid volume below the physiological range, remains a common yet understudied obstetric condition within environmental health research. Previous studies have found that oligohydramnios significantly increases the risk of small-for-gestational-age (SGA) infants, fetal distress, neonatal death, and long-term neurodevelopmental, endocrinal and cardiovascular deficits.^3–6^ Oligohydramnios is clinically important because it disrupts fetal growth and intrauterine homeostasis^3,7^ and often necessitates urgent delivery.^8^ Notably, isolated oligohydramnios, which occurs in otherwise healthy pregnancies without identifiable maternal or fetal disorders, accounts for approximately 50%–70% of cases^7^ and is increasingly recognized as part of the placental-insufficiency spectrum.^9^ Its abrupt onset, unclear etiology, and lack of reliable early-warning indicators pose significant challenges for clinical management and highlight the need for identifying modifiable environmental risk factors.^3,7,8,10,11^

Amniotic fluid homeostasis is governed by a set of interconnected physiological pathways, including placental perfusion, intramembranous transport across the fetal membranes, and fetal renal and swallowing function, and the relative contribution of each pathway changes across gestation.^3,7^ Early pregnancy depends more heavily on membrane-mediated fluid exchange, whereas mid- to late pregnancy increasingly relies on placental perfusion and fetal urine production.^3,7^ Perturbations in these processes, particularly placental malperfusion,^9,12^ membrane inflammation or oxidative injury,^13,14^ and dysregulation of aquaporin-mediated transport,^15,16^ are increasingly implicated in the pathophysiology of isolated oligohydramnios. Growing evidence shows that common ambient air pollutants affect these same biological systems: particulate and traffic-related pollutants impair placental vascular remodeling and induce inflammation and oxidative stress^2,17,18^; and experimental data suggest impacts on maternal or fetal renal perfusion.^19,20^ Together, these mechanistic findings support a biologically plausible hypothesis that prenatal air pollution exposure may interfere with gestational stage-specific regulation of amniotic fluid volume and increase the risk of isolated oligohydramnios.

Beyond these mechanistic considerations, it is important to situate this research within the broader global landscape of ambient air pollution. Global assessments indicated that many regions worldwide continued to experience concentrations far exceeding the 2021 World Health Organization (WHO) Air Quality Guidelines (PM_2_._5_: 5 μg/m^3^; NO_2_: 10 μg/m^3^).^21,22^ More than 90% of the global population is exposed to air pollution levels above WHO guidelines, with particularly high multipollutant burdens in South Asia and parts of Africa for PM_2_._5_, in the Middle East and highly urbanized regions for NO_2_, and in the Middle East, South Asia, and East Asia for seasonally elevated O_3_. These patterns highlight that high-level, mixed-pollutant exposures remain common across many regions worldwide.^21^ These regional patterns highlight that high-level, multipollutant exposure remains the predominant reality for large populations, especially in low- and middle-income settings. Yet, most existing epidemiologic studies on pregnancy and air pollution have been conducted in North America and Europe,^23^ where ambient concentrations are substantially lower, leaving an important evidence gap regarding maternal susceptibility and pollutant mixture effects at higher exposure ranges.

Despite extensive research on air pollution and other obstetric outcomes, virtually no studies have examined its relationship with oligohydramnios, particularly isolated oligohydramnios, and none have assessed mixture effects or identified gestationally specific susceptibility windows. This represents a major evidence gap, given that real-world ambient air pollution consists of complex mixtures of correlated pollutants whose effects cannot be disentangled using traditional single-pollutant models. Understanding which pollutants, at which gestational periods, exert the greatest influence is crucial for developing practical prevention strategies for a condition that currently lacks early-warning indicators. The weighted quantile sum (WQS) regression framework provides a structured approach for evaluating highly correlated pollutant mixtures, yet its conventional formulation does not account for the time-varying nature of prenatal exposures.^24,25^ The recently developed lagged WQS (lWQS) model overcomes this limitation by simultaneously quantifying overall mixture effects and estimating weekly pollutant-specific contributions across gestation, making it uniquely suited for pregnancy research.^26,27^ To date, no studies have applied lWQS, or any other mixture model, to assess week-specific effects of prenatal pollutant mixtures on any obstetric outcome, representing both a methodological and epidemiologic gap.

Building on these knowledge gaps, we conducted a retrospective cohort study based on births from a large tertiary hospital in northwestern China to systematically examine the association between prenatal exposure to ambient air pollution mixtures and the risk of isolated oligohydramnios. Due to its developed industry, terrain, geographical location, and winter heating, this city is exposed to high air pollution conditions. We applied the lWQS model to identify dominant pollutants and critical time windows, and used restricted cubic spline (RCS) modeling to explore concentration–effect relationships and protective thresholds. This study aims to fill an important knowledge gap by providing epidemiological evidence for the mechanistic links between pollutant exposure, fetal membranes, and amniotic fluid regulation, and by offering spatiotemporal targets and exposure benchmarks to inform gestation-stage-specific environmental health interventions.

## Methods

### Study design and population

This was a hospital-based retrospective cohort study that included 10,856 singleton live births delivered at the First Affiliated Hospital of Xi’an Jiaotong University between 2015 and 2019. To align with the study objective of evaluating isolated oligohydramnios, we restricted the analytic cohort to term pregnancies. This restriction minimized exposure misclassification, because the date of birth was used as a proxy for the diagnosis date, an approach valid primarily in term pregnancies where diagnosis and delivery occur in close temporal proximity.^7^ Furthermore, it could avoid the influence of severe obstetric complications that frequently underlie preterm delivery.

Conditions known to directly reduce amniotic fluid volume were excluded, including premature rupture of membranes (defined as rupture before labor onset), fetal structural abnormalities (renal or cardiac anomalies), and intrauterine infection (amnionitis). These proximal etiologic factors are incompatible with the definition of isolated oligohydramnios. We also excluded pregnancies with serious maternal or fetal complications (e.g., hypertensive disorders, pregestational diabetes, immune disease, fetal growth restriction—representative of poor placental function) that could indirectly alter amniotic fluid volume or obscure environmental effects. Polyhydramnios cases and records with missing key variables were likewise removed.

**Figure 1.**
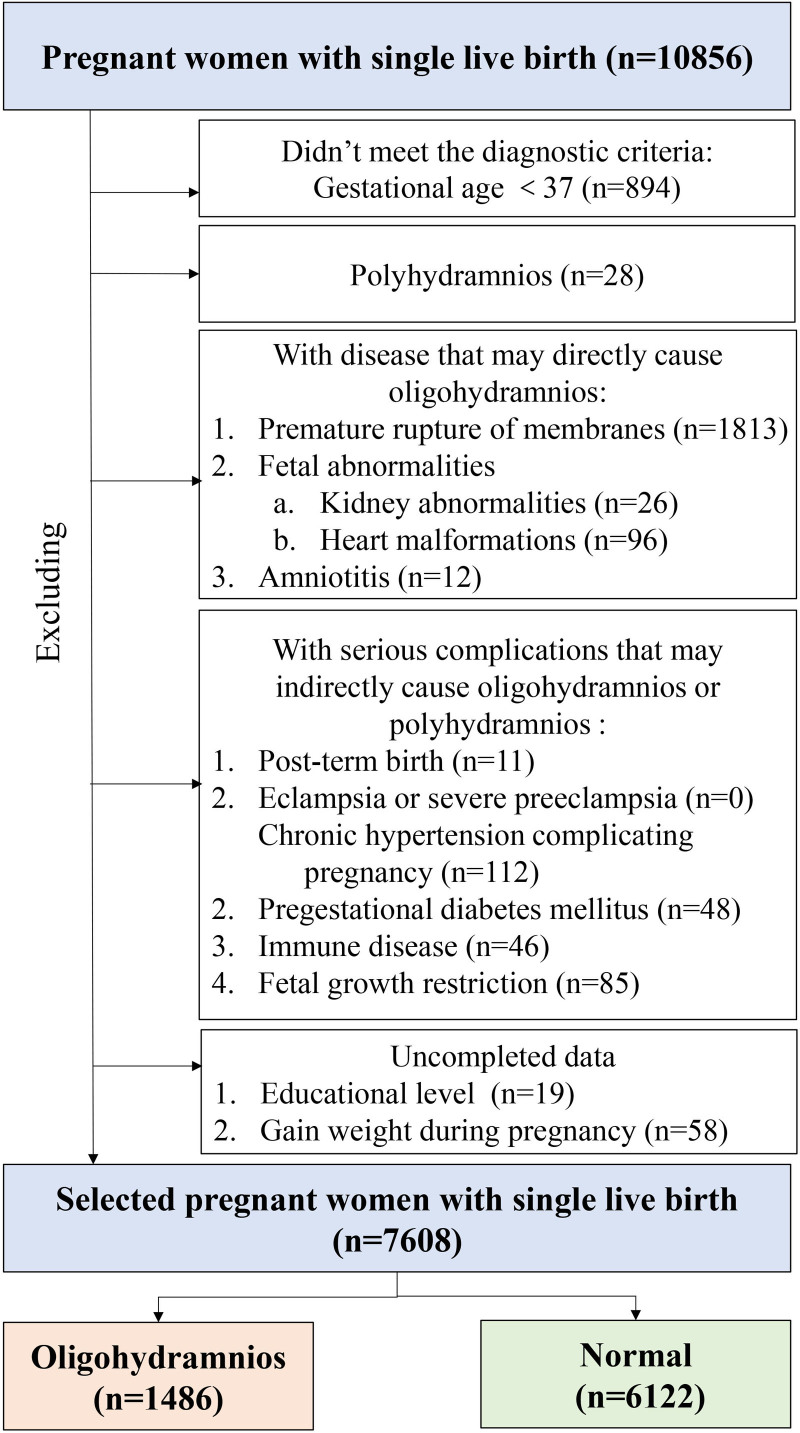
Flow chart for inclusion and exclusion of participants in the final analysis.

Ultimately, as shown in Figure 1, 7,608 pregnant women were included in the analysis, of whom 1,486 had term isolated oligohydramnios, and the remaining 6,122 were normal controls. All maternal and neonatal data were retrospectively extracted from electronic medical records. As this study involved secondary analysis of anonymized retrospective data, informed consent from participants was not required. The study protocol was approved by the Medical Ethics Committee of the First Affiliated Hospital of Xi’an Jiaotong University (Approval No. XJTU1AF2019LSK-088).

### Outcome definition

Oligohydramnios was defined as a maximum vertical pocket of ≤2 cm or an amniotic fluid index (of ≤5 cm on late pregnancy ultrasound.^28^ Due to the lack of precise gestational age at diagnosis in electronic medical records, the gestational age at delivery was used as a proxy, following previous studies,^10,29^ as the diagnosis typically occurs within days before delivery and is closely tied to its timing, particularly in term pregnancies.^7^

Gestational age was calculated by adjusting the last menstrual period (LMP) using crown-rump length from the 11–13 gestational week ultrasound, which is the standard approach in obstetric practice. Importantly, biological conception occurs around the end of gestational week 2 (LMP + 14 days).^30^ This distinction is essential for subsequent exposure assessment and interpretation of time-sensitive analyses. In all exposure calculations, we therefore aligned biological pregnancy onset to conception. However, for clarity, clinical relevance, and public communication, we continue to present gestational timing using standard LMP-based gestational-age labels.

### Exposure assessment

Air pollution data (PM_2_._5_, PM_10_, SO_2_, O_3_, CO, and NO_2_) were obtained from 13 monitoring stations in Xi’an (2014–2019), managed by the Ministry of Ecology and Environment (http://www.mee.gov.cn/). The spatial distribution of participants and air quality monitoring stations is provided in Supplementary Figure S1; https://links.lww.com/EE/A410. The daily missing rate was below 0.5%, and missing values were filled using linear interpolation, followed by seasonal adjustment. Meteorological data, including daily average temperature and relative humidity, were retrieved from the China Meteorological Data Network (http://data.cma.cn/) using records from local meteorological stations in Xi’an.

We used the inverse distance weighting method to estimate individual-level air pollution exposure. This method assumes that spatially closer locations have more similar pollution levels. Daily concentrations were calculated based on the geocoded addresses of pregnant women, weighted using data from nearby monitoring stations—an approach widely used in epidemiological studies.^31,32^

We calculated several exposure metrics to support multilevel analyses. Because biological conception occurs near the end of gestational week 2 (LMP + 14 days), actual embryonic and fetal exposure begins at the start of gestational week 3. The exposure metrics were specifically computed as follows: (1) average concentrations over the entire pregnancy (3 week to delivery); (2) trimester-specific means for first (3–13 weeks), second (14–27 weeks), and third (28 week to delivery) trimester, also called early, mid, and late pregnancy; (3) weekly specific means concentrations (3–37 weeks); (4) average temperature and relative humidity during each time window.

### Covariates

Variables were retrospectively collected from medical records and categorized as follows: (1) sociodemographic: maternal age, ethnicity, occupation, neonatal sex, parity, season of conception, year of conception(reflecting temporal trends in air pollution; see Supplementary Figure S3; https://links.lww.com/EE/A410), delivery mode, education level, and GWG; (2) disease-related: SGA, gestational hypertensive disorders (excluding eclampsia, severe preeclampsia, and chronic hypertension, which were exclusion criteria), gestational diabetes, and intrauterine fetal distress; (3) meteorological: daily average temperature and relative humidity.

A directed acyclic graph (Supplementary Figure S2; https://links.lww.com/EE/A410) was used to guide covariate selection. Based on the temporal ordering and presumed causal structure, gestational weight gain (GWG), gestational hypertensive disorders (HDP), and gestational diabetes (GDM) were classified as mediators rather than confounders. The minimal sufficient adjustment set included maternal age at delivery, occupation, education level, season of conception, year of conception, and daily average temperature and relative humidity. These variables were therefore adjusted for in all primary analyses.

### Statistical analysis

The aim of this study was to systematically assess the effects of mixed air pollutant exposure during pregnancy on oligohydramnios, and to further identify critical mixed exposure periods and protection thresholds to guide the development of protection strategies.

#### Single pollutant and mixture effects analyses

To preliminarily assess the independent effects of prenatal air pollution exposure on oligohydramnios, we first evaluated the association for each single pollutant (PM_2_._5_, PM_10_, NO_2_, SO_2_, CO, and O_3_) across the entire pregnancy. Linearity of the exposure–response relationships was examined using generalized additive models. The generalized additive model results indicated approximately linear relationships for PM_2_._5_, NO_2_, and O_3_, whereas PM_10_, SO_2_, and CO exhibited clear nonlinear patterns (Supplementary Figure S4 and Supplementary Table S2; https://links.lww.com/EE/A410). To avoid imposing linearity assumptions, all single-pollutant exposures were categorized into quartiles, and logistic regression models were fitted using the lowest quartile (Q1) as the reference group. Results were reported as odds ratios (ORs) and 95% confidence intervals (CIs), representing the contrast between higher exposure quartiles and Q1 (i.e., Q2–Q4 vs. Q1).

To account for joint exposure scenarios, we constructed a WQS regression model^24,33^ to evaluate the overall effect of mixed pollutants on oligohydramnios. The six pollutants were categorized into four quartiles based on concentration, and a WQS index was generated as the weighted sum of pollutant-specific quantile scores. The weights were constrained to be negative or positive depending on the assumed direction of the mixture effect, and were normalized to sum to 1. Exposure indices were fitted in the training set (40%) and evaluated in the validation set (60%) via a bootstrap procedure. The model was run 1,000 times in both positive-direction and negative-direction models, yielding mean pollutant weights and the joint effects for both directions. The resulting WQS ORs represent the change in risk associated with a one-unit increase in the WQS index. To complementarily determine whether mixed air pollutants exhibited different susceptibility windows across pregnancy, separate WQS models were developed for early, mid, and late pregnancy.

#### Identification of mixed critical windows and air pollutants weights

The lWQS model is a scalable method for analyzing mixed exposures, where pollutant concentrations at different time points are treated as predictors to explore lagged effects on health outcomes.^26^ Unlike traditional WQS, lWQS constructs separate WQS indices for each lag point and applies penalized modeling along with stability selection to address multicollinearity and variable selection in high-dimensional temporal data.

In this study, we constructed lWQS models covering gestational weeks 3–37 to assess critical exposure weeks. Results were evaluated using 1,000 bootstrap resamples and were expressed as ORs and 95%CIs for each week. A critical window was defined as a time point where the beta coefficient was significantly nonzero and appeared consistently in >60% of bootstrap iterations. lWQS also provides average pollutant weights within each window, enabling identification of dominant pollutants for dose–response modeling and threshold estimation.

#### 3. Concentration–effect modeling

Based on key gestational weeks identified by the lWQS models, we selected representative pollutants based on their weights to construct RCS models. These models explored the nonlinear concentration–effect relationships between pollutant concentrations and the risk of oligohydramnios within major exposure windows. Each RCS model was based on a multivariable logistic regression framework with three knots, and results were presented as concentration–effect curves (ORs) with 95% CI, relative to zero pollutant concentration.

Correspondingly, because weekly exposures exhibit substantially greater short-term variability, their percentile cut points are not directly comparable to percentiles derived from pregnancy-average exposure metrics. Thresholds and percentile positions were determined using the exposure distribution of the weekly concentrations, rather than pregnancy-average concentrations.

#### Sensitivity analyses

To assess the robustness of our findings, we conducted several sensitivity analyses.

First, at the whole-pregnancy exposure level, we applied formal counterfactual-based mediation analysis to quantify the mediating roles of GWG, HDP, and GDM in the associations between single pollutants and oligohydramnios.

Second, we evaluated potential effect modification by key maternal characteristics. Stratified analyses were performed for both single-pollutant and multipollutant exposures according to maternal age at delivery, educational level, ethnicity, occupation, and parity, to identify particularly vulnerable subgroups.

In addition, to evaluate the robustness and generalizability of the results, we conducted the following subgroup analyses: (1) analyses including preterm delivery (PTD) and finally participants, and analyses restricted to preterm populations, to examine the influence of gestational-age exclusion criteria on our estimates; (2) analyses restricted to pregnant women without any comorbidities, to minimize potential confounding by underlying maternal medical conditions; and (3) analyses restricted to participants residing within 10 km of an air quality monitoring station, to reduce exposure estimation error and validate the accuracy of the inverse distance weighting method. For all these subgroups, we repeated single-pollutant logistic regression and multipollutant WQS analyses for the entire pregnancy.

Finally, we constructed single-pollutant distributed lag nonlinear models (DLNMs) for each pollutant to assess whether the identified key gestational weeks were consistently associated with risk.

All statistical analyses were conducted using the R software (version 4.3.2; https://www.r-project.org/), with the “gwqs,” “lwqs,” “rms,” and “dlmn” packages. Statistical significance was defined as a two-tailed *P* value <0.05.

## Results

### Population characteristics and distribution of exposure

Table 1 presents the baseline characteristics of the study population. Compared with the control group, the oligohydramnios group had a younger mean age (29.60 vs. 30.32 years, *P* < 0.001), higher GWG (15.65 vs. 15.23 kg, *P* = 0.001), and a markedly higher proportion of primigravidas (75.1% vs. 61.5%, *P* < 0.001). In addition, the incidences of fetal distress (17.5% vs. 13.0%, *P* < 0.001) and SGA infants (11.4% vs. 5.2%, *P* < 0.001) were significantly higher. Significant differences were also observed in the mode and season of cenception.

**Table 1.** Baseline characteristics of the study participants (N = 7,608)

Covariates	Categories	Total (N =7,608)	Normal (N = 6,122)	Oligohydramnios (N = 1,486)		*P* value
**Maternal age (years**)		**30.18 (3.87**)	**30.32 (3.90**)	**29.60 (3.67**)		**<0.001**
Gestational age at delivery (weeks)		39.00 [38.00, 40.00]	39.00 [38.00, 40.00]	39.00 [39.00, 40.00]		0.071
**Gain weight (kg**)		**15.31 (4.47**)	**15.23 (4.44**)	**15.65 (4.55**)		**0.001**
Ethnicity	Han	7,549 (99.2)	6,075 (99.2)	1,474 (99.2)		1.000
	Minorities	59 (0.8)	47 (0.8)	12 (0.8)		
Educational level (years)	≤9	6,575 (86.4)	5,292 (86.4)	1,283 (86.3)		0.320
	10~12	562 (7.4)	442 (7.2)	120 (8.1)		
	>12	471 (6.2)	388 (6.3)	83 (5.6)		
Occupation	Farmer	151 (2.0)	119 (1.9)	32 (2.2)		0.201
	Worker	3,647 (47.9)	2,971 (48.5)	676 (45.5)		
	Others	2,856 (37.5)	2,277 (37.2)	579 (39.0)		
	None	954 (12.5)	755 (12.3)	199 (13.4)		
Gender	Male	3,933 (51.7)	3,179 (51.9)	754 (50.7)		0.428
	Female	3,675 (48.3)	2,943 (48.1)	732 (49.3)		
**Parity**	**Primipara**	**4,882 (64.2**)	**3,766 (61.5**)	**1,116 (75.1**)		**<0.001**
	**Multipara**	**2,726 (35.8**)	**2,356 (38.5**)	**370 (24.9**)		
**Delivery mode**	**Vaginal**	**4,007 (52.7**)	**3,189 (52.1**)	**818 (55.0**)		**0.044**
	**Cesarean**	**3,601 (47.3**)	**2,933 (47.9**)	**668 (45.0**)		
**Season of conception**	**Spring**	**1,885(24.8**)	**1,528 (25.0**)	**357 (24.0**)		**0.001**
	**Summer**	**1,891(24.9**)	**1,527 (24.9**)	**364 (24.5**)		
	**Autumn**	**1,921(25.2**)	**1,525 (24.9**)	**396 (26.7**)		
	**Winter**	**1,911(25.1**)	**1,542 (25.2**)	**369 (24.8**)		
Hypertensive disorders of pregnancy	Yes	296 (3.9)	247 (4.0)	49 (3.3)		0.214
	No	7,312 (96.1)	5,875 (96.0)	1,437 (96.7)		
Gestational diabetes	Yes	473 (6.2)	392 (6.4)	81 (5.5)		0.192
	No	7,135 (93.8)	5,730 (93.6)	1,405 (94.5)		
**Intrauterine fetal distress**	**Yes**	**1,057 (13.9**)	**797 (13.0**)	**260 (17.5**)		**<0.001**
	**No**	**6,551 (86.1**)	**5,325 (87.0**)	**1,226 (82.5**)		
**Small for gestation**	**Yes**	**489 (6.4**)	**319 (5.2**)	**170 (11.4**)		**<0.001**
	**No**	**7,119 (93.6**)	**5,803 (94.8**)	**1,316 (88.6**)		
**Birth weight (g**)		**33,70.35 (397.54**)	**3,395.65 (394.64**)	**3,266.10 (392.55**)	**<0.001**	

Bold values indicate statistical significance (p < 0.05). Values are presented as number and percentage [N (%)] for categorical variables or mean ± standard deviation (SD) for continuous variables.

Categorical variables were compared using the chi-square or Fisher’s exact test; continuous variables were compared using independent-sample *t* test or Wilcoxon rank-sum test, as appropriate.

As shown in Supplementary Table S1; https://links.lww.com/EE/A410, median concentrations of PM_2.5_ (64.07 μg/m^3^), PM_10_ (126.73 μg/m^3^), and NO_2_ (52.96 μg/m^3^), even their minimum values (33.95, 71.01, and 23.01 μg/m^3^, respectively), all substantially exceeded the corresponding WHO Global Air Quality Guidelines for long-term exposure (10, 15, and 10 μg/m^3^, respectively). For O_3_, we calculated the pregnancy-average of the daily maximum 8-hour mean concentration. The WHO guideline for O_3_ long-term exposure, defined as the highest 6-month running average of the daily maximum 8-hour mean concentration (60 μg/m^3^), falls within the first quartile (Q1: 45.91–75.39 μg/m^3^) of the exposure distribution in our study population. The broad interquartile ranges observed across pollutants indicate marked interindividual variability in exposure. Supplementary Figure S5; https://links.lww.com/EE/A410 further illustrates the Spearman correlations among pollutants and meteorological variables: PM_2.5_ and PM_10_ were highly correlated (*r* ≈ 0.8), NO_2_ showed moderate correlations with PM pollutants, while O_3_ was negatively correlated with other pollutants.

### Associations between maternal air pollution exposure and oligohydramnios risk

Table 2 displays the associations between air pollution exposure during pregnancy and the risk of isolated oligohydramnios. PM_2_._5_, NO_2_, and O_3_ demonstrated increasing risk across exposure quartiles, with significant effects observed in the high category [PM_2_._5_ Q4: OR = 1.274 (95% CI 1.008, 1.611); NO_2_ Q3: OR = 1.324 (1.107, 1.583) and Q4: OR = 1.381 (1.149, 1.661); O_3_ Q3: OR = 1.370 (1.102, 1.703) and Q4: OR = 1.494 (1.120, 1.994)]. For **SO**_**2**_ and **CO**, only moderate exposure (Q2) was associated with significantly elevated risks [SO_2_ Q2: OR = 1.318 (1.108, 1.567); CO Q2: OR = 1.204 (1.013, 1.432)], while higher quartiles did not retain significance. PM_10_ showed no consistent or statistically significant associations across exposure categories.

**Table 2.** Associations between maternal exposure to air pollutants and risk of oligohydramnios

Variables	Single-pollutant effect [OR (95% CI)]^a^				Mixture effect^b^			
					Positive direction^c^		Negative direction^d^	
	Q1	Q2	Q3	Q4	Weight^e^	OR (95% CI)^b^	Weight^e^	OR (95% CI)^b^
PM_2.5_	-	1.014 (0.828, 1.242)	1.237 (0.971, 1.576)	**1.274 (1.008, 1.611**)	17.28%		0.29%	
PM_10_	-	1.152 (0.966, 1.374)	0.911 (0.746, 1.113)	1.016 (0.812, 1.270)	2.78%		30.90%	
NO_2_	-	1.167 (0.977, 1.393)	**1.324 (1.107, 1.583**)	**1.381 (1.149, 1.661**)	47.05%	**1.204**	9.36%	0.952
O_3_	-	1.156 (0.971, 1.376)	**1.370 (1.102, 1.703**)	**1.494 (1.120, 1.994**)	21.33%	**(1.049, 1.285**)	19.32%	(0.885, 1.285)
SO_2_	-	**1.318 (1.108, 1.567**)	1.131 (0.942, 1.358)	0.929 (0.770, 1.120)	5.40%		10.00%	
CO	-	**1.204 (1.013, 1.432**)	1.169 (0.976, 1.401)	0.875 (0.721, 1.062)	6.16%		39.50%	

Bold values indicate statistical significance (p < 0.05). All models were adjusted for maternal age at delivery, occupation, education level, season of conception, year of conception, temperature, and relative humidity.

a Single-pollutant effects: odds ratios (ORs) represent the contrast between quartiles of each pollutant (Q2–Q4 vs. Q1 as the reference).

b Mixture effect: weighted quantile sum (WQS) regression models estimate the overall joint effect of the pollutant mixture. The WQS OR reflects the change in oligohydramnios risk associated with a one-unit increase in the WQS index.

c Positive direction indicates that the WQS model was fitted under a positive constraint, where higher joint exposure (higher WQS index) is associated with higher odds of oligohydramnios.

d Negative mixture indicates that the WQS model was fitted under a negative constraint, where higher joint exposure is associated with lower odds.

e Weights indicate the relative contribution of each pollutant to the WQS index under the specified model direction and sum to 100% within each direction.

Multipollutant exposure analyses stratified by gestational stages (Table 3) revealed distinct trimester-specific effects of pollutant mixtures on the risk of oligohydramnios: (1) in first trimester, a significant positive joint effect was observed (OR = 1.220, 95% CI 1.076, 1.437), primarily driven by O_3_ (49.96%) and NO_2_ (20.71%); (2) in second trimester, the joint effect was not significant (OR = 1.206, 95% CI 0.917, 1.587); (3) in third trimester, the association was strongest (OR = 1.360, 95% CI, 1.107, 1.459), with O_3_ and NO_2_ contributing most, 39.16% and 25.50%. In contrast, no significant protective effects were observed in the negative-direction WQS model.

**Table 3.** Mixture effects of maternal exposure to multiple air pollutants on oligohydramnios by pregnancy trimester

Period	Variables	Weight^a^	Mixture OR (95% CI)^b^
First trimester	**Positive direction** ^c^		
	**O**_**3**_	**49.96%**	
	**NO**_**2**_	**20.71%**	
	**CO**	**14.07%**	**1.220 (1.076, 1.437**)
	**SO**_**2**_	**9.53%**	
	**PM**_**10**_	**5.59%**	
	**PM**_**2.5**_	**0.14%**	
	Negative direction^d^		
	PM_2.5_	67.59%	
	O_3_	24.49%	
	PM_10_	4.84%	1.076 (0.693, 1.145)
	NO_2_	1.66%	
	CO	1.40%	
	SO_2_	0.02%	
Second trimester	Positive direction^c^		
	NO_2_	35.99%	
	O_3_	30.34%	
	SO_2_	20.47%	1.206 (0.917, 1.587)
	PM_10_	7.13%	
	CO	4.19%	
	PM_2.5_	1.87%	
	Negative direction^d^		
	PM_2.5_	47.97%	
	CO	30.47%	
	PM_10_	10.13%	1.154 (0.875, 1.520)
	O_3_	8.03%	
	SO_2_	3.30%	
	NO_2_	0.10%	
Third trimester	**Positive direction** ^c^		
	**O**_**3**_	**39.16%**	
	**NO**_**2**_	**25.50%**	
	**PM**_**2.5**_	**25.86%**	**1.360 (1.107, 1.459**)
	**CO**	**7.81%**	
	**PM**_**10**_	**1.97%**	
	**SO**_**2**_	**0.70%**	
	Negative direction^d^		
	SO_2_	63.42%	
	PM_10_	30.02%	
	O_3_	6.49%	0.946 (0.755, 1.188)
	PM_2.5_	0.02%	
	CO	0.02%	
	NO_2_	0.02%	

Bold values indicate statistical significance (p < 0.05). All models were adjusted for maternal age at delivery, occupation, education level, season of conception, year of conception, temperature, and relative humidity.

a Weights indicate the relative contribution of each pollutant to the WQS index under the specified model direction and sum to 100% within each direction.

b The WQS OR reflects the change in oligohydramnios risk associated with a one-unit increase in the WQS index.

c Positive direction indicates that the WQS model was fitted under a positive constraint, where higher joint exposure (higher WQS index) is associated with higher odds of oligohydramnios.

d Negative mixture indicates that the WQS model was fitted under a negative constraint, where higher joint exposure is associated with lower odds.

### Identification of susceptible windows and dominant pollutants

**Figure 2.**
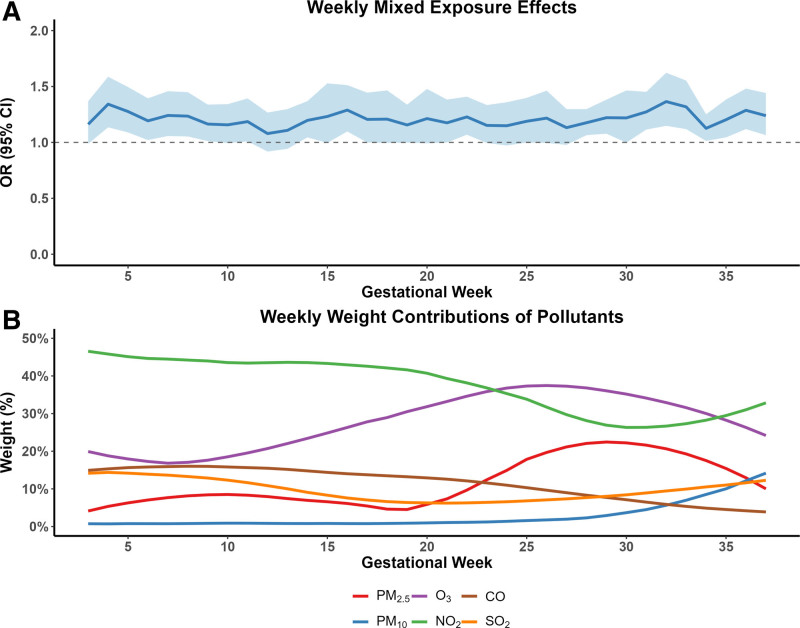
Weekly effects of maternal exposure to air pollutant mixtures on oligohydramnios risk. (A) Lagged associations between weekly maternal exposure to air pollutant mixtures and oligohydramnios. (B) Mean weights of single pollutants at each gestational week. The WQS OR reflects the change in oligohydramnios risk associated with a one-unit increase in the WQS index. Adjusted for maternal age at delivery, occupation, education level, season of conception, year of conception, temperature, and relative humidity.

In the long-term exposure analysis (Figure 2A, Supplementary Table S3, and Supplementary Figure S6; https://links.lww.com/EE/A410), the lWQS model revealed elevated risk of oligohydramnios from several gestational weeks, mainly in the first and third trimesters. Distinct peaks in these three trimesters, week 4 (OR = 1.342, 95% CI 1.135, 1.587), week 16 (OR = 1.289, 95% CI 1.098, 1.512), and week 32 (OR = 1.365, 95% CI 1.148, 1.623), respectively. Regarding pollutant contributions (Figure 2B, Supplementary Figure S6; https://links.lww.com/EE/A410), O_3_ and NO_2_ were the two dominant contributors to risk across most gestational weeks. O_3_ consistently showed the highest mean weights from early to mid-pregnancy (weeks 3–23, weighted 36.8%–46.5%), while NO_2_ gradually increased in influence, surpassing O_3_ around weeks 24–34, weighted 30.0R%–36.8%. PM_2.5_ showed a notable rise in contribution during the third trimester. In contrast, PM_10_, SO_2_, and CO maintained relatively stable and lower contributions throughout pregnancy.

### Estimation of preventive thresholds via concentration–response curves

**Figure 3.**
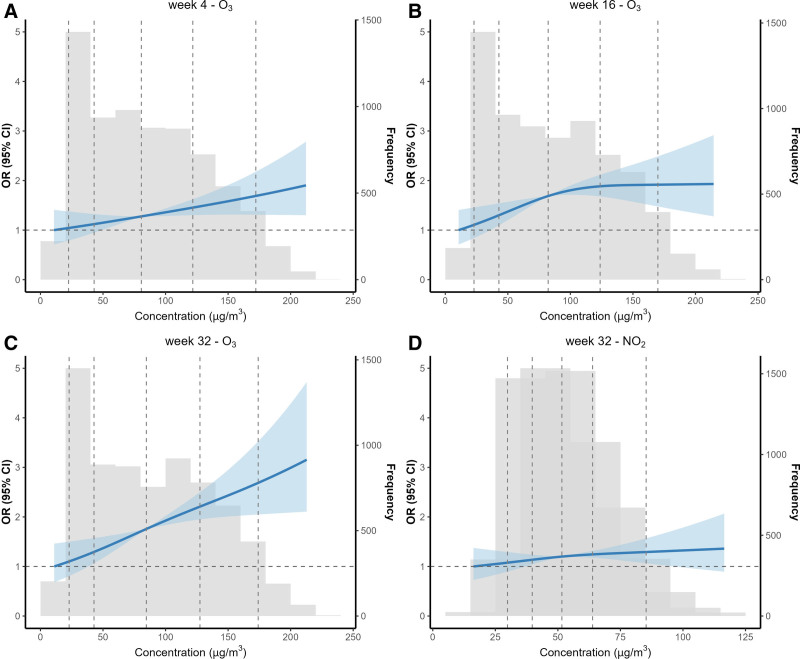
Concentration–response curves for selected pollutants in representative gestational weeks. Restricted cubic spline models adjusted for maternal age at delivery, occupation, education level, season of conception, year of conception, temperature, and relative humidity. Vertical dashed lines indicate the 5th, 25th, 50th, 75th, and 95th percentiles of the weekly pollutant concentrations, as weekly exposures exhibit substantially greater short-term variability; their percentile cut points are not directly comparable to percentiles derived from pregnancy-average exposure metrics.

To explore stage-specific protective thresholds, we selected key exposure windows identified by the lWQS model—weeks 4, 16, and 32—representing early, mid and late pregnancy, and acute exposure. Concentration–effect curves were constructed for the corresponding dominant pollutants (O_3_ and/or NO_2_) to estimate preventive thresholds (Figure 3).

In week 4, O_3_ exposure was associated with a near-linear increase in oligohydramnios risk. A significant threshold was observed at 49.28 μg/m^3^. In week 16, the concentration–effect curve for O_3_ showed a significant risk threshold at 36.28 μg/m^3^. In week 32, both O_3_ and NO_2_ were significantly associated with risk, with gradually increasing concentration–effect curves. The thresholds were 37.40 μg/m^3^ for O_3_ and 37.41 μg/m^3^ for NO_2_.

### Sensitivity analyses

In mediation analyses, total effects comparing exposure quartiles (Q2–Q4 vs. Q1) were decomposed into natural direct (NDE) and indirect (NIE) effects. Across all pollutants, NIE estimates were consistently close to null, with 95% CIs overlapping one (Supplementary Figure S7; https://links.lww.com/EE/A410), and the proportion mediated was small—generally within ±10% with wide intervals crossing zero (Supplementary Figure S8; https://links.lww.com/EE/A410).

Stratified analyses showed broadly consistent associations across maternal demographic and socioeconomic subgroups (Supplementary Figure 9; https://links.lww.com/EE/A410). In the single-pollutant models, effect estimates were similar across categories of maternal age, education, ethnicity, occupation, and parity, with overlapping CIs and no clear evidence of strong subgroup differences. A one-unit increase in the WQS index was statistically significant among women aged <35 years, those with ≤9 years of education, Han ethnicity, and those employed in other or unemployed occupations, whereas other strata showed nonsignificant but directionally similar associations.

Subgroup analyses confirmed the robustness of the findings. Repeating the analyses in combined preterm and term populations, as well as after excluding pregnancies with maternal or fetal complications, produced effect estimates similar in magnitude and direction to the primary results across all six pollutants. Mixture effects from WQS regression were likewise stable. Restricting the cohort to women residing within 10 km of a monitoring station yielded comparable or slightly stronger associations.

DLNM analyses aligned with these findings and reinforced the gestational susceptibility patterns detected in the lWQS models. Linear DLNMs for PM_2_._5_, NO_2_, and O_3_ indicated modest early- to mid-pregnancy effects with clearer risk elevations in late gestation (approximately weeks 30–34). Nonlinear DLNMs for PM_10_, SO_2_, and CO showed similar late-pregnancy vulnerability regions in the exposure–lag response surfaces (Supplementary Figure S10; https://links.lww.com/EE/A410).

## Discussion

This is the first study to explore the association between air pollution and oligohydramnios and to systematically examine exposure-informed prevention strategies. We identified critical exposure windows, with late pregnancy and short-term periods before diagnosis being especially vulnerable. O_3_ and NO_2_ emerged as dominant risk factors during these periods. Notably, adverse effects were observed even at pollutant concentrations below conventional safety thresholds, underscoring potential health risks from low-level exposures. These findings provide new insights into the temporal and pollutant-specific dynamics of environmental risk and support the need for further investigation into gestation-specific environmental exposure effects.

### Interpretation of observed associations with air pollution exposure

In single-pollutant analyses, exposures to PM_2.5_, NO_2_, and O_3_ were all positively associated with oligohydramnios after full adjustment in the high category (PM_2_._5_ Q4, NO_2_ Q3 and Q4, O_3_ Q3 and Q4), while SO_2_ and CO, only moderate exposure (Q2) was associated with significantly elevated risks. In addition, the WQS model further revealed the relative contributions of pollutants throughout pregnancy, with PM_2.5_, NO_2_, and O_3_ accounting for more than 80%. Given the substantial collinearity among pollutants in this setting, single-pollutant models may obscure the underlying exposure dimensions that drive health effects. This consistency across modeling approaches suggests that these pollutants represent the primary drivers of the observed associations rather than artifacts of confounding by co-pollutants. In contrast, SO_2_ and CO, which demonstrated elevated risks only at moderate exposure levels in single-pollutant models, contributed minimally to the mixture index, indicating their limited influence when co-exposure patterns are considered. The null findings for PM_10_ in both modeling frameworks further support its negligible independent contribution in this population.

As this is the first study to explore the association between maternal exposure to ambient air pollution and oligohydramnios, there are no directly comparable findings in the literature. However, substantial evidence links prenatal air pollution exposure to a range of adverse pregnancy outcomes marked by placental dysfunction, including preeclampsia, fetal growth restriction, preterm birth, stillbirth, and low birth weight. In a recent systematic review summarizing more than 30 million births in the United States, PM_2_._5_ and O_3_ were repeatedly identified as dominant contributors to reduced birthweight and preterm delivery, with effect estimates comparable to those observed in our study.^34^ Mixture analyses from large population-based cohorts also highlight NO_2_ and O_3_ as major drivers of pregnancy complications: Jiang et al. reported that PM_2_._5_, CO, and O_3_ jointly increased the risk of hypertensive disorders of pregnancy, with O_3_ contributing strongly to the mixture effect.^35^ Sun et al. demonstrated that NO_2_ was the leading contributor to increased gestational diabetes risk in multipollutant models.^36^ Jiao et al. showed that the overall positive mixture effect on premature rupture of membranes was primarily driven by O_3_.^37^ These studies collectively support that photochemical oxidants and traffic-related pollutants often dominate mixture effects in pregnancy and tend to act through pathways involving placental stress, vascular dysregulation, and membrane integrity.

Importantly, since oligohydramnios is also closely tied to placental health, some researchers, such as Miremberg,^9^ have proposed classifying it within the spectrum of placental insufficiency. The convergence of evidence across different but pathophysiologically related outcomes thus reinforces the plausibility of our findings: pollutants that consistently emerge as dominant contributors in mixture models for placental-mediated complications in other cohorts (notably NO_2_ and O_3_) are the same pollutants that carry the highest weights in our WQS analysis.

In the preliminary exploration of time sensitivity with trimester-stratified WQS models, we observed meaningful differences across pregnancy stages. The joint effects remained significant in both the first and third trimesters, with the strongest effect in late pregnancy. These findings motivated the use of the lWQS model to further identify week-specific susceptibility. Importantly, because biological conception occurs near the end of gestational week 2, the relevant exposure period for early pregnancy begins around gestational week 3 onward.^30^ This distinction applies not only to the calculation of whole-pregnancy and early-pregnancy average exposures but also to the interpretation of the week-specific sensitive windows derived from the lWQS analysis.

### Identifying sensitive exposure windows and key pollutants

The lWQS analysis revealed that the susceptibility to mixed air pollution exposure was not constant across gestation but exhibited gestational stage-specific peaks, with the strongest elevations in early and late pregnancy and a secondary rise in mid-pregnancy. These windows align well with known transitions in amniotic fluid regulation: early pregnancy relies predominantly on membrane-mediated fluid exchange, whereas mid- to late pregnancy increasingly depends on placental perfusion and fetal urine production.^3,7^ The time-varying pollutant weights further clarified which mixture components were most influential at each window. O_3_ dominated early to mid-pregnancy, NO_2_ became increasingly influential in mid-to late pregnancy, and PM_2_._5_ showed a modest rise in contribution toward the third trimester—patterns that may reflect the alignment of pollutant-specific toxicity profiles with evolving physiological pathways of amniotic fluid homeostasis. In contrast, PM_10_, SO_2_, and CO contributed minimally across gestation, suggesting a comparatively limited role in the overall mixture effect.

Although no previous study has examined sensitive exposure windows for oligohydramnios specifically, the temporal structure identified in our analysis is broadly consistent with week- or trimester-specific associations reported for other placental-mediated complications. For gestational diabetes, large cohort studies have highlighted early to mid-pregnancy as a critical period: in Ontario, O_3_ exposure from the late first through the second trimester showed the strongest associations,^38^ while in Western New York, NO_2_ exhibited sensitive windows around the periconceptional and early gestational months.^39^ For fetal growth outcomes, weekly-resolution DLNMs have likewise detected discrete vulnerability periods, including NO_2_-related reductions in fetal size during weeks 5–12^40^ and O_3_-associated risks of preterm birth and low birth weight during early, mid, and late gestation.^41^

Taken together, these studies support a recurring pattern in which early placentation, mid-gestation vascular remodeling, and late-pregnancy fetal growth and fluid regulation^42^ constitute common susceptibility windows across diverse perinatal outcomes, while photochemical oxidants and traffic-related fine particles repeatedly emerge as dominant mixture components. This broader evidence base closely mirrors the temporal peaks and pollutant-specific contributions of O_3_, NO_2_, and PM_2_._5_ identified in our oligohydramnios analysis. These time-dependent shifts emphasize that both the underlying biological processes and the dominant toxicity drivers evolve over the course of gestation, highlighting the importance of pollutant-specific and time-specific public health interventions.

### Implications of concentration–response analyses for risk control

The concentration–response analyses provide several insights with direct implications for gestational risk management. For the peak-risk windows identified by the lWQS models—week 4 (O_3_), week 16 (O_3_), and week 32 (O_3_ and NO_2_)—we estimated week-specific preventive thresholds to inform potential exposure guidance during pregnancy. The threshold for O_3_ was 49.28 μg/m^3^ at gestational week 4, and 36.28 μg/m^3^ at week 16. At week 32, the thresholds were 37.40 μg/m^3^ for O_3_ and 37.41 μg/m^3^ for NO_2_.

Because these thresholds were derived from weekly exposure distributions, which represent short-term exposure metrics, we compared them with the WHO 2021 Air Quality Guidelines for short-term exposures—O_3_ (100 μg/m^3^, 8-hour), NO_2_ (25 μg/m^3^, 24-hour)^22^。Notably, the O_3_ thresholds identified for weeks 4 and 16 were far below the WHO short-term guideline. This suggests that measurable increases in risk may occur even within exposure levels considered acceptable under existing short-term standards. In contrast, the NO_2_ threshold at week 32 exceeded the WHO-recommended short-term value, indicating that late pregnancy may represent a period of heightened susceptibility to NO_2_.

Together, these findings raise important questions about whether current short-term air quality benchmarks adequately protect pregnant women—particularly in regions with high baseline pollution. Rather than contradicting WHO guidance, these results underscore the need to consider pregnancy-specific susceptibility and to refine exposure thresholds for maternal–fetal health.

### Potential mechanism

Evidence shows that placental development is accompanied by rising oxidative and nitrosative stress, and that disturbances in placental perfusion and redox balance influence multiple pregnancy outcomes.^2,43^ Early pregnancy depends primarily on membrane-mediated fluid exchange, mid-gestation on placental vascular remodeling, and late pregnancy on fetal urine production and adequate placental perfusion.^42^ Although direct mechanistic evidence linking these pollutants to oligohydramnios is still limited, the combined oxidative, vascular, and epigenetic perturbations described in the literature offer a biologically plausible pathway through which mixed air pollutants may contribute to reduced amniotic fluid volume in a gestational-stage-specific manner.

During early pregnancy, ozone’s well-established oxidant properties may be relevant to the vulnerability of trophoblast invasion and membrane-mediated fluid exchange at this stage. O_3_ generates secondary reactive species and systemic inflammatory signals and has been linked to increased maternal and cord inflammatory markers and reduced global DNA methylation.^2^ Oxidative or inflammatory perturbations caused by O_3_ may plausibly disrupt early placental–membrane integrity and reduce fluid-exchange efficiency. For mid-pregnancy, NO_2_ and O_3_ may evaluate the risk of oligohydramnios by placental vascular remodeling. They were reported to be associated with nitrosative stress, reduced placental mitochondrial DNA content, altered DNA methylation, and impaired uteroplacental Doppler indices, and induce placental hypoxia, inflammation, and premature senescence.^2,44–46^ By late pregnancy, when amniotic fluid volume depends largely on fetal urine output and adequate fetoplacental perfusion, NO_2_ again dominated the pollutant mixture, consistent with its documented effects on placental hypoxia pathways and vascular dysfunction. Reduced placental oxygen and nutrient delivery could plausibly diminish fetal renal perfusion and urine production. In addition, the modest rise in PM_2_._5_ contribution during this stage may reflect cumulative particulate effects, as PM_2_._5_ has been associated with placental oxidative DNA damage, mitochondrial dysfunction, and hypomethylation of growth-related genes.^2,43^

Together, these mechanisms highlight the placenta and its circulatory environment as central targets through which oxidant gases and fine particles may alter fluid homeostasis, offering a biologically plausible explanation for the stage-specific susceptibility observed in our analyses.

### Sensitivity Analyses

Multiple sensitivity analyses demonstrated that the observed associations were robust across analytical specifications and population subsets.

Additionally, mediation analyses suggest that, within our study population, the relationship between air pollution and oligohydramnios risk was driven primarily by direct effects rather than by maternal metabolic or hypertensive conditions. This may partly reflect our strict exclusion criteria, which removed pregnancies with severe forms of HDP and GDM, thereby minimizing confounding by strong clinical determinants of amniotic fluid abnormalities. Among women with relatively mild metabolic or blood pressure profiles, these pathways may not constitute major mediating mechanisms. Further mechanistic clarification will likely require prospective cohorts with repeated maternal health assessments and biomarker-based phenotyping.

Meanwhile, although the WQS mixture effect reached statistical significance only in certain strata (e.g., <35 years, ≤9 years of education), the effect directions and magnitudes were similar across groups, indicating limited evidence of true heterogeneity.

### Strengths and limitations

This study has several notable strengths. First, it is the first to systematically investigate the association of air pollution and oligohydramnios—a clinically significant but long-overlooked obstetric complication associated with adverse perinatal outcomes. Second, the use of a standardized inpatient birth cohort ensured high data quality, and internal validity was strengthened through strict inclusion criteria and multiple sensitivity analyses. Third, we integrated WQS, lWQS, and RCS models to establish a pollutant time–dose risk framework. This approach not only enabled the identification of dominant pollutants and critical gestational windows but also estimated exposure thresholds with potential preventive relevance. In particular, the novel application of lWQS to assess time-specific effects of pollutant mixtures provides both methodological innovation and actionable insight for targeted prenatal environmental interventions.

Nonetheless, this study has some limitations. First, exposure estimates were based on fixed-site monitoring data, which may not fully capture individual-level variations. Although sensitivity analyses reduced this concern. Second, as data were drawn from a single medical center, the findings may not be fully generalizable to populations. Third, oligohydramnios was referenced to the timing of delivery rather than the exact date of diagnosis; although restricting the population to term deliveries minimized potential exposure misclassification, more precise clinical timing is needed in future studies. Fourth, while the strict inclusion and exclusion criteria minimized major confounding from severe maternal conditions and allowed us to focus on the air pollution-oligohydramnios pathway, this approach may have masked more complex interactions or mediation structures. Finally, the absence of biological or placental markers limits mechanistic interpretation. Taken together, future research incorporating refined personal-level exposure assessment, precise clinical timing of oligohydramnios onset, longitudinal maternal biomarkers, placental evaluations, repeated clinical measurements, and data from diverse geographic regions will be essential to elucidate underlying mechanisms, strengthen causal inference, and enhance the generalizability of these findings.

## Conclusion

This study is the first to identify a significant association between maternal exposure to mixed ambient air pollutants and oligohydramnios. Leveraging a standardized inpatient birth cohort and an integrated WQS–lWQS–RCS framework, we uncovered critical exposure windows in the early and late pregnancy. O_3_ and NO_2_ were consistently dominant contributors, with the threshold of O_3_ at 49.28 μg/m^3^ for the early pregnancy, O_3_ at around 37.00 μg/m^3^ for the mid- and late pregnancy, and NO_2_ at 37.41 μg/m^3^ for the late pregnancy. These findings underscore the need for gestation- and pollutant-specific prevention strategies and offer actionable exposure thresholds to inform public health interventions.

## Conflicts of interest statement

The authors declare that they have no conflicts of interest with regard to the content of this report.

## ACKNOWLEDGMENTS

*We sincerely thank all staff members from the Department of Obstetrics and Gynecology and the Information Center of the First Affiliated Hospital of Xi’an Jiaotong University for their support in data retrieval and patient management. We are also grateful to the personnel at the Xi’an Air Pollution Monitoring Station, Xi’an Meteorological Monitoring Station, the Ministry of Ecology and Environment of China, and the China Meteorological Data Network for providing essential environmental data and technical assistance.*

## Supplementary Material
