# Supplementary material for: Critical windows and risk thresholds of prenatal mixed air pollutant exposure for oligohydramnios: Evidence from a population‑based study
Source: Environ Epidemiol. 2026 Feb 12;10(2):e454. doi: 10.1097/EE9.0000000000000454 (PMC12904356; doi:10.1097/EE9.0000000000000454)
Supplement: Supplementary file 1 [file ee9-10-e454-s001.pdf]

Manuscript number: EE-D-25-00104

Manuscript title: Critical Windows and Risk Thresholds of Prenatal Mixed Air Pollutant Exposure for Oligohydramnios: Evidence from a Population-Based Study

Supplementary Materials

Supplementary Table S1. Distribution of air pollutant concentrations in our cohort compared with the 2021 WHO Air Quality Guidelines.

Supplementary Table S2. Quantitative judgment indicators and judgment results of linear/nonlinear air pollutants by generalized additive models.

Supplementary Table S3. Weekly lagged effects of air pollutants mixture on oligohydramnios risk.

Supplementary Table S4. The associations between air pollution and oligohydramnios -sensitivity analysis of premature and term delivery population.

Supplementary Table S5. The associations between air pollution and oligohydramnios - sensitivity analysis of women without complications and women live within 10km for monitor station.

Supplementary Figure S1. Spatial Distribution of participants and Air Quality Monitoring Stations in Xi'an.

Supplementary Figure S2. Directed acyclic graph.

Supplementary Figure S3. Temporal trends of pollutant concentration from 2015 to 2019.

Supplementary Figure S4. Linear/nonlinear correlation graph between air pollutants and oligohydramnios by generalized additive models.

Supplementary Figure S5. Correlations among air pollutants and meteorological variables.

Supplementary Figure S6. Mean weights of air pollutants for weekly lagged mixture effects and on oligohydramnios risk.

Supplementary Figure S7. The effects of gestational weight gain, gestational hypertensive disorders, and gestational diabetes on the relationship between air pollution and oligohydramnios-mediation analyses.

Supplementary Figure S8. The proportion mediated values of gestational weight gain, gestational

hypertensive disorders, and gestational diabetes on the relationship between air pollution and oligohydramnios-mediation analyses.

Supplementary Figure S9. Comparison of the effects of air pollution and oligohydramnios among different subgroups.

Supplementary Figure S10. The weekly impact of a single pollutant on oligohydramnios calculated by DLNM - pollutant as a continuous variable.

Supplementary Table S1. Distribution of air pollutant concentrations in our cohort compared with the 2021 WHO Air Quality Guidelines.

Variables	Mean	SD	percentile					IQR	AQG
			Min	P25	P50	P75	Max		
$PM_{2.5}$ (μg/m^3)	64.51	13.58	33.95	55.16	64.07	71.17	118.11	16.02	5 (annual)
PM_{10} (μg/m^3)	126.43	19.92	71.01	112.76	126.73	139.72	181.76	68.72	15 (annual)
NO_2 (μg/m^3)	53.37	6.68	23.01	49.29	52.96	57.53	71.36	8.24	10 (annual)
O_3 (μg/m^3)	87.76	15.89	45.91	75.39	87.35	102.05	128.07	26.66	60 (peak season*)
SO_2 (μg/m^3)	17.49	6.50	5.32	12.16	17.10	21.86	36.59	9.70	40 (24-hour)
CO (mg/m^3)	1.43	0.34	0.66	1.14	1.45	1.69	2.29	0.56	4 (24-hour)

* Defined as the average of daily maximum 8-hour means during the six consecutive months with the highest six-month running-average O_3 concentration.

Abbreviations: WHO = World Health Organization.

Since no annual AQG values are available for SO_2 and CO, their 24-hour AQG values are presented herein.

Supplementary Table S2. Quantitative judgment indicators and judgment results of linear/nonlinear air pollutants by generalized additive models.

Pollutant	Edf	P-value for s(x)	ΔAIC*	P-value for LRT	Relationship type
$PM_{2.5}$	1.98	0.023	-0.30	0.093	Linear
PM_{10}	4.14	0.021	8.25	0.001	Nonlinear
NO_2	1.01	＜0.001	-0.02	0.016	Linear
O_3	1.25	＜0.001	-0.38	0.123	Linear
SO_2	3.37	＜0.001	22.17	＜0.001	Nonlinear
CO	3.16	＜0.001	19.82	＜0.001	Nonlinear

Abbreviations: LRT = Likelihood ratio test.

*ΔAIC= AIC (Linear)- AIC (Nonlinear)

Supplementary Table S3. Weekly lagged effects of air pollutants mixture on oligohydramnios risk.

Gestational week	OR(95%CI)	Gestational week	OR(95%CI)	Gestational week	OR(95%CI)
3	1.162(0.987,1.368)	15	1.232(0.996,1.528)	27	1.132(0.978,1.298)
**4**	**1.342(1.135,1.587)**	**16**	**1.289(1.098,1.512)**	**28**	**1.176(1.065,1.298)**
**5**	**1.276(1.089,1.495)**	17	1.206(0.999,1.445)	**29**	**1.221(1.078,1.382)**
**6**	**1.193(1.021,1.394)**	18	1.208(0.995,1.465)	30	1.219(0.998,1.465)
**7**	**1.241(1.057,1.458)**	19	1.156(0.998,1.338)	**31**	**1.273(1.115,1.452)**
**8**	**1.235(1.053,1.449)**	20	1.213(0.992,1.478)	**32**	**1.365(1.148,1.623)**
**9**	**1.164(1.012,1.338)**	21	1.175(0.999,1.383)	**33**	**1.318(1.119,1.553)**
10	1.157(0.993,1.342)	**22**	**1.228(1.069,1.409)**	**34**	**1.126(1.013,1.251)**
**11**	**1.186(1.009,1.393)**	23	1.152(0.994,1.334)	**35**	**1.203(1.045,1.383)**
12	1.079(0.918,1.267)	24	1.149(0.973,1.359)	**36**	**1.287(1.119,1.481)**
13	1.108(0.943,1.298)	25	1.189(0.998,1.398)	**37**	**1.239(1.065,1.442)**
**14**	**1.197(1.045,1.369)**	26	1.217(0.995,1.463)		

The WQS OR reflects the change in oligohydramnios risk associated with a one-unit increase in the WQS index.

Adjusted for maternal age at delivery, occupation, education level, season of conception, year of conception, temperature and relative humidity.

Supplementary Table S4. The associations between air pollution and oligohydramnios -sensitivity analysis of premature and term delivery population.

Variables	Quantile	OR(95%CI)		
		**Premature and term delivery population**	**Term delivery population**	**Premature delivery population**
$PM_{2.5}$[a]	Q1	-	-	-
	Q2	0.968(0.797,1.176)	1.014(0.828,1.242)	1.403(0.448,4.388)
	Q3	1.22(0.968,1.536)	1.237(0.971,1.576)	1.537(0.317,7.453)
	Q4	**1.297(1.034,1.627)**	**1.274(1.008,1.611)**	1.167(0.222,6.122)
PM_{10}[a]	Q1	-	-	-
	Q2	**1.255(1.059,1.487)**	1.152(0.966,1.374)	1.595(0.556,4.576)
	Q3	1.063(0.875,1.292)	0.911(0.746,1.113)	1.872(0.536,6.540)
	Q4	1.161(0.930,1.448)	1.016(0.812,1.270)	0.616(0.116,3.268)
NO_2[a]	Q1	-	-	-
	Q2	1.125(0.948,1.335)	1.167(0.977,1.393)	0.581(0.223,1.514)
	Q3	**1.314(1.105,1.562)**	**1.324(1.107,1.583)**	0.340(0.110,1.053)
	Q4	**1.424(1.189,1.706)**	**1.381(1.149,1.661)**	0.767(0.241,2.441)
O_3[a]	Q1	-	-	-
	Q2	1.175(0.993,1.392)	1.156(0.971,1.376)	1.629(0.500,5.309)
	Q3	**1.384(1.118,1.714)**	**1.370(1.102,1.703)**	1.060(0.215,5.231)
	Q4	**1.479(1.117,1.958)**	**1.494(1.120,1.994)**	2.086(0.261,16.704)
SO_2[a]	Q1	-	-	-
	Q2	**1.342(1.136,1.584)**	**1.318(1.108,1.567)**	2.597(0.978,6.892)
	Q3	**1.207(1.012,1.440)**	1.131(0.942,1.358)	2.014(0.655,6.193)
	Q4	0.980(0.816,1.177)	0.929(0.770,1.120)	0.600(0.156,2.301)
CO[a]	Q1	-	-	-
	Q2	**1.257(1.064,1.485)**	**1.204(1.013,1.432)**	1.979(0.779,5.028)
	Q3	**1.223(1.025,1.460)**	1.169(0.976,1.401)	1.069(0.348,3.283)
	Q4	0.955(0.79,1.1540)	0.875(0.721,1.062)	0.606(0.169,2.168)
Mixture[b]	-	**1.124(1.016,1.320)**	**1.204(1.049,1.285)**	0.897(0.397,1.957)

NOTE: All models were adjusted for maternal age at delivery, occupation, education level, season of conception, year of conception, temperature, and relative humidity.

a Single-pollutant effects: Odds ratios (ORs) represent the contrast between quartiles of each pollutant (Q2–Q4 vs. Q1 as the reference).

b Mixture effect: Weighted quantile sum (WQS) regression models estimate the overall joint effect of the pollutant mixture. The WQS OR reflects the change in oligohydramnios risk associated with a one-unit increase in the WQS index.

Supplementary Table S5. The associations between air pollution and oligohydramnios - sensitivity analysis of women without complications and women live within 10km for monitor station.

Variables	Quantile	OR(95%CI)	
		**Women without complications**	**Women lived within 10km for monitor station**
$PM_{2.5}$ [a]	Q1	-	-
	Q2	1.013(0.793,1.294)	1.062(0.859,1.314)
	Q3	1.234(0.920,1.655)	1.270(0.984,1.639)
	Q4	1.215(0.915,1.614)	**1.304(1.018,1.670)**
PM_{10} [a]	Q1	-	-
	Q2	1.179(0.954,1.457)	1.127(0.937,1.356)
	Q3	0.913(0.717,1.164)	0.903(0.731,1.114)
	Q4	1.047(0.800,1.371)	0.998(0.790,1.262)
NO_2 [a]	Q1	-	-
	Q2	1.206(0.971,1.499)	**1.260(1.041,1.525)**
	Q3	**1.512(1.219,1.875)**	**1.409(1.162,1.709)**
	Q4	**1.399(1.117,1.752)**	**1.436(1.177,1.752)**
O_3 [a]	Q1	-	-
	Q2	1.155(0.936,1.425)	1.149(0.955,1.382)
	Q3	**1.441(1.110,1.871)**	**1.337(1.065,1.680)**
	Q4	**1.756(1.241,2.484)**	**1.421(1.052,1.920)**
SO_2 [a]	Q1	-	-
	Q2	**1.356(1.100,1.671)**	**1.320(1.102,1.581)**
	Q3	1.094(0.876,1.367)	1.053(0.868,1.278)
	Q4	0.955(0.762,1.197)	0.904(0.741,1.102)
CO [a]	Q1	-	-
	Q2	**1.233(1.001,1.519)**	1.173(0.979,1.404)
	Q3	1.101(0.882,1.374)	1.141(0.943,1.379)
	Q4	0.877(0.695,1.106)	0.829(0.675,1.018)
Mixture [b]	-	**1.197(1.033,1.280)**	**1.255(1.124,1.439)**

NOTE: All models were adjusted for maternal age at delivery, occupation, education level, season of conception, year of conception, temperature, and relative humidity.

[a] Single-pollutant effects: Odds ratios (ORs) represent the contrast between quartiles of each pollutant (Q2–Q4 vs. Q1 as the reference).

[b] Mixture effect: Weighted quantile sum (WQS) regression models estimate the overall joint effect of the pollutant mixture. The WQS OR reflects the change in oligohydramnios risk associated with a one-unit increase in the WQS index.

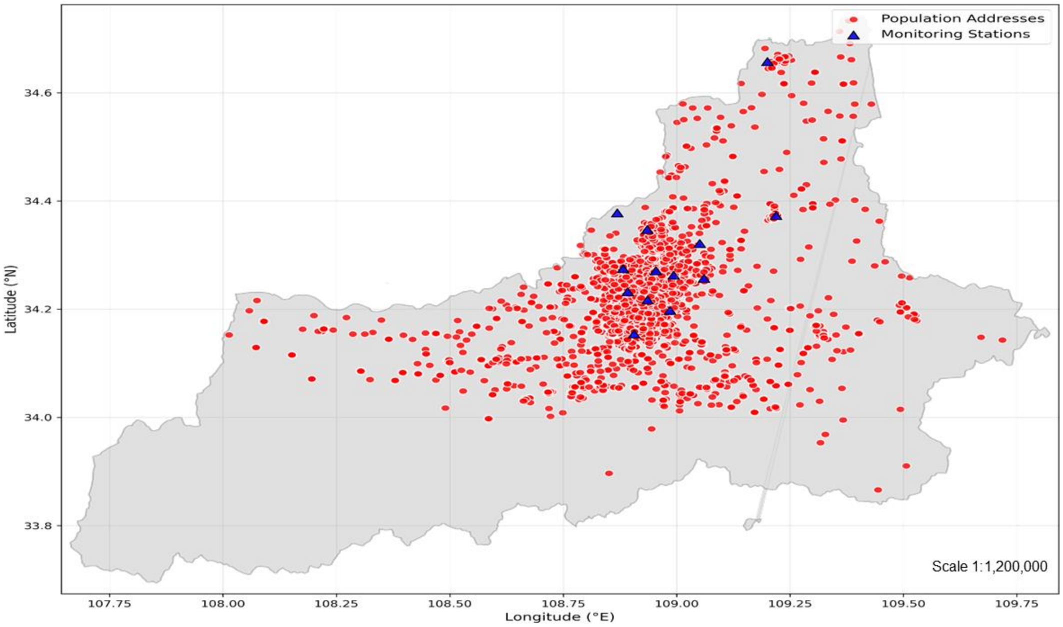

Supplementary Figure S1. Spatial Distribution of participants and Air Quality Monitoring Stations in Xi'an.

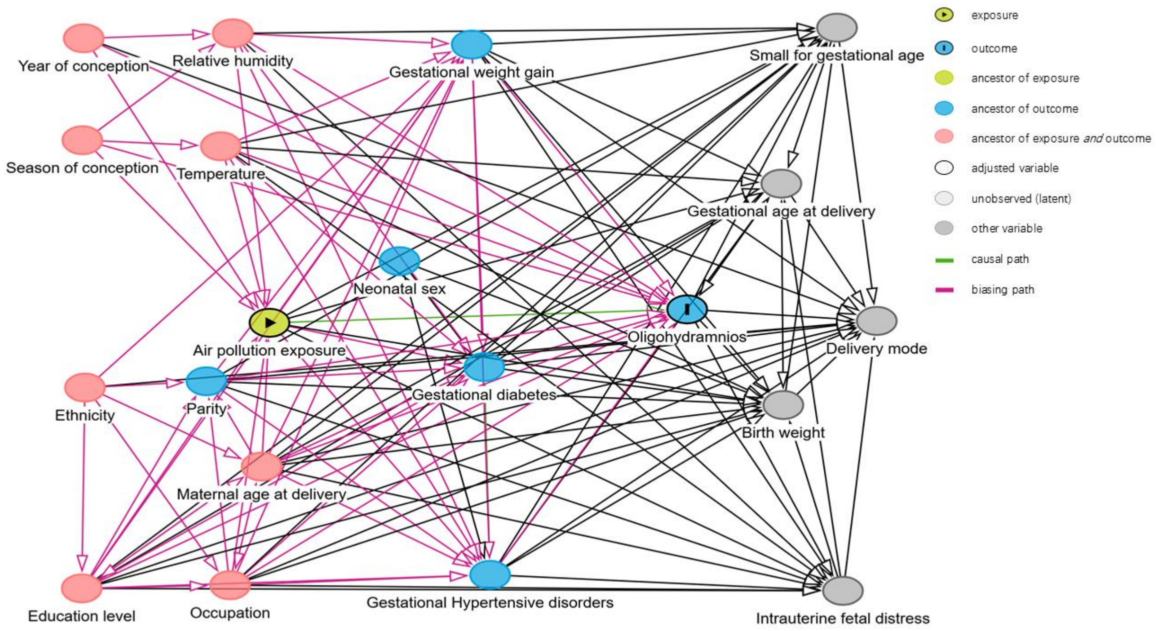

Supplementary Figure S2. Directed acyclic graph.

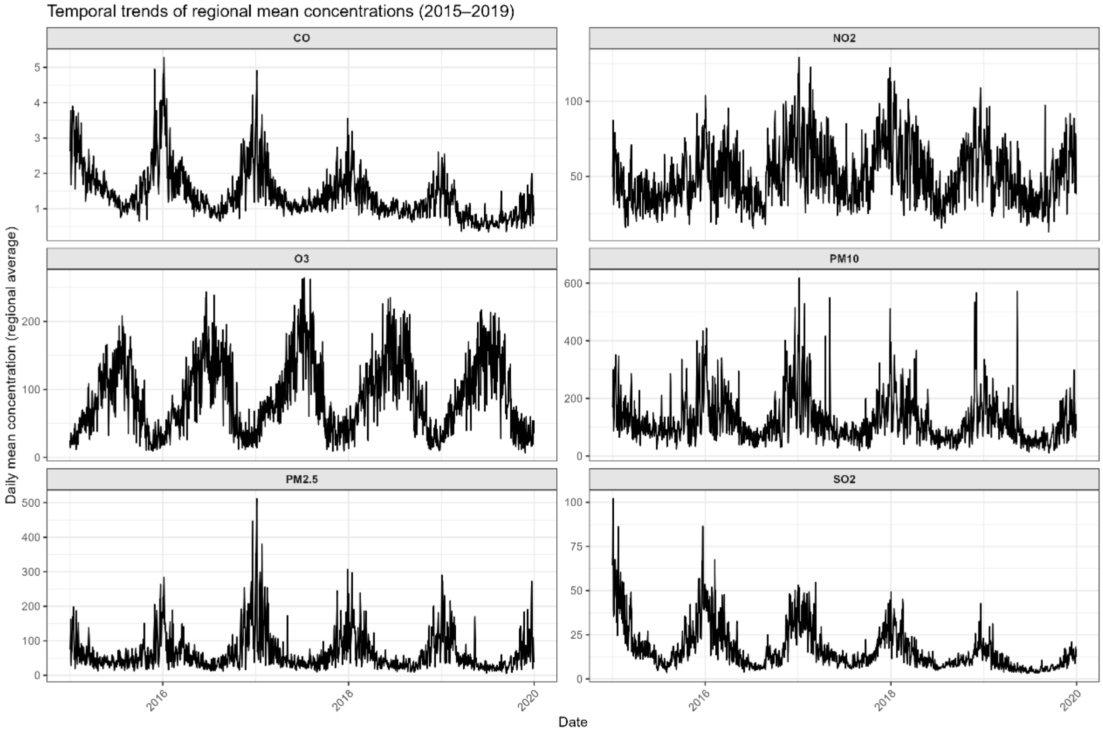

Supplementary Figure S3. Temporal trends of pollutant concentration from 2015 to 2019.

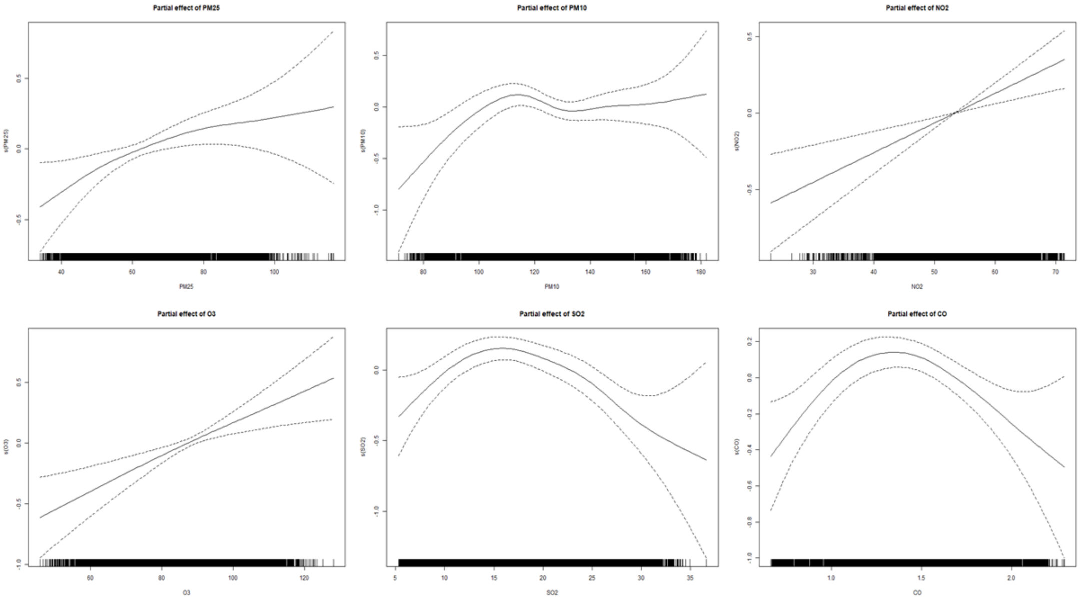

Supplementary Figure S4. Linear/nonlinear correlation graph between air pollutants and oligohydramnios by generalized additive models. All models were adjusted for maternal age at delivery, occupation, education level, season of conception, year of conception, temperature, and relative humidity.

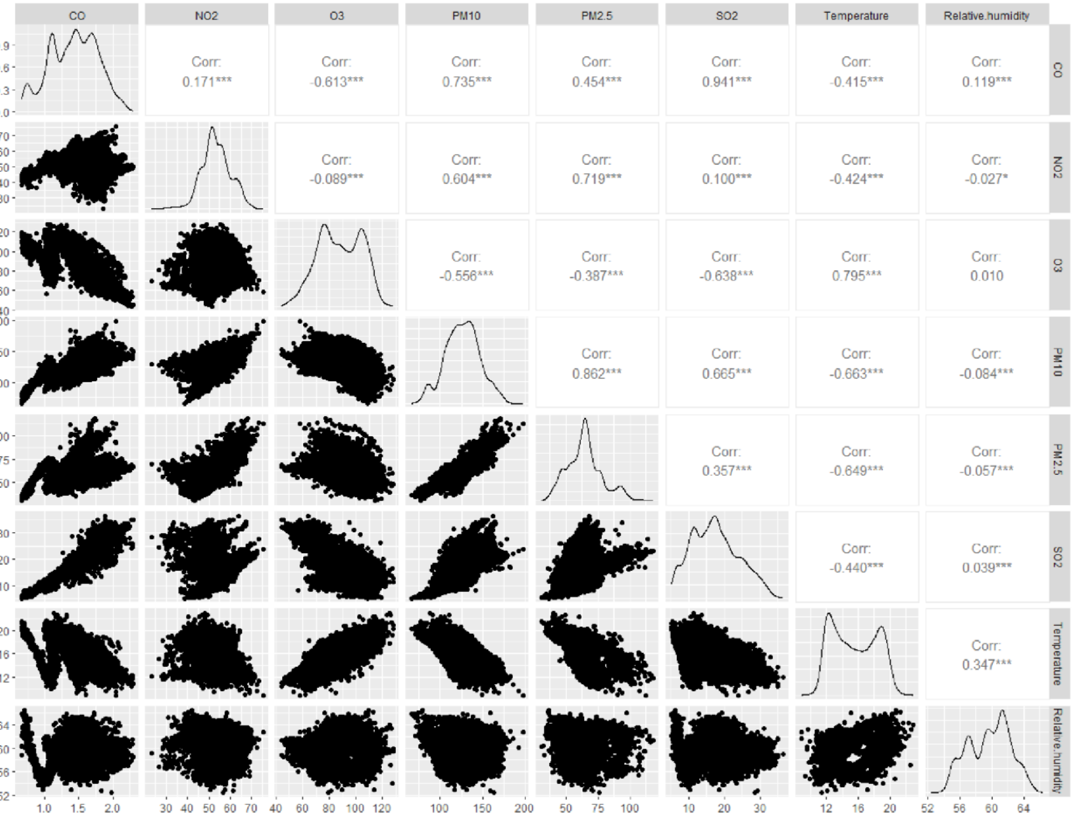

Supplementary Figure S5. Correlations among air pollutants and meteorological variables.

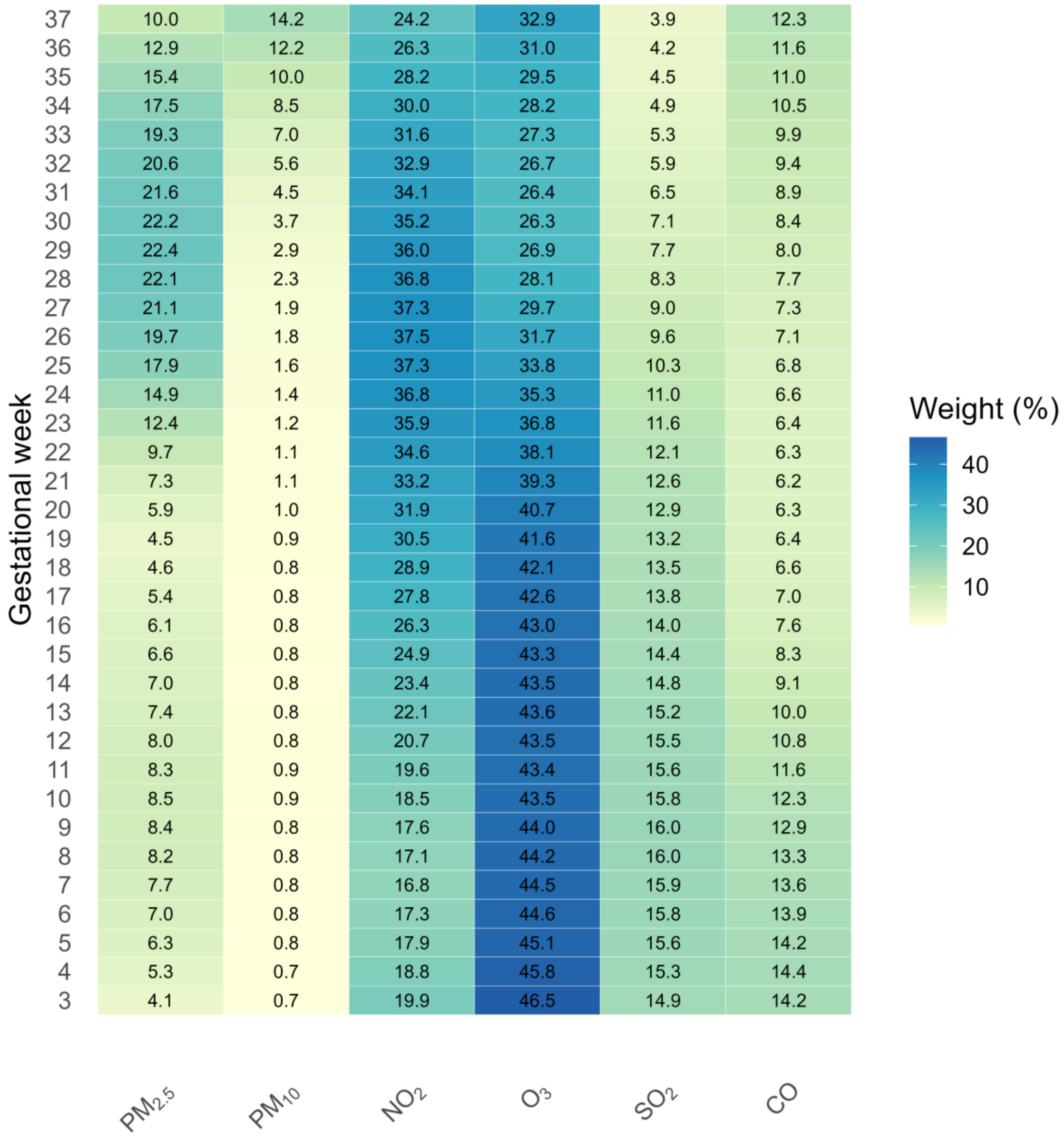

Supplementary Figure S6. Mean weights of air pollutants for weekly lagged mixture effects and on oligohydramnios risk. Weight results estimated by lagged weighted quantile sum (lWQS) model. Weights indicate the relative contribution of each pollutant to the WQS index under the specified model direction and sum to 100% within each direction.

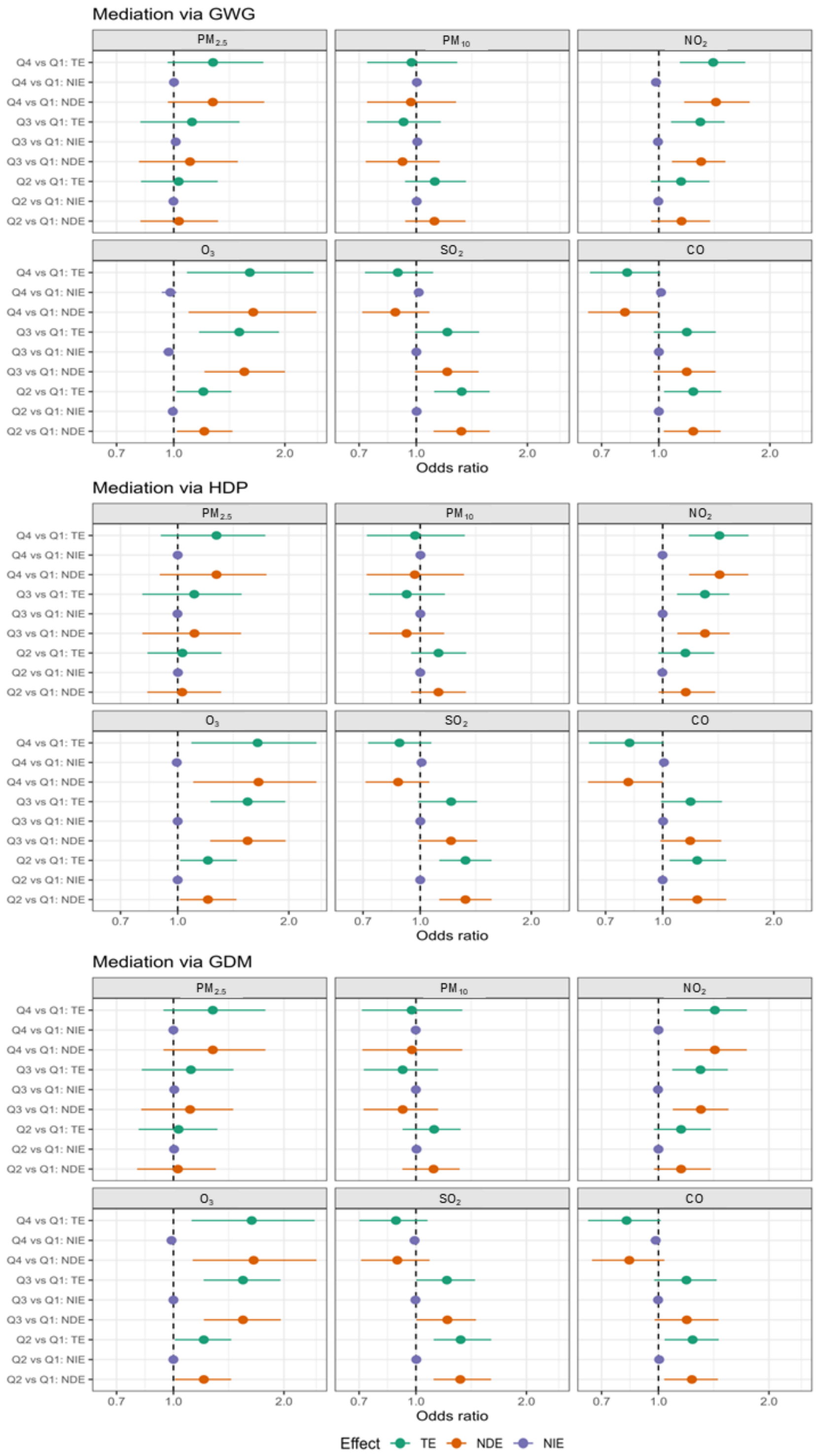

Supplementary Figure S7. The effects of gestational weight gain, gestational hypertensive disorders, and gestational diabetes on the relationship between air pollution and oligohydramnios-mediation analyses. TE: total effect, NDE: natural direct effect, NIE: natural indirect effect, GWG: gestational weight gain, HDP: gestational hypertensive disorders, GDM: gestational diabetes.

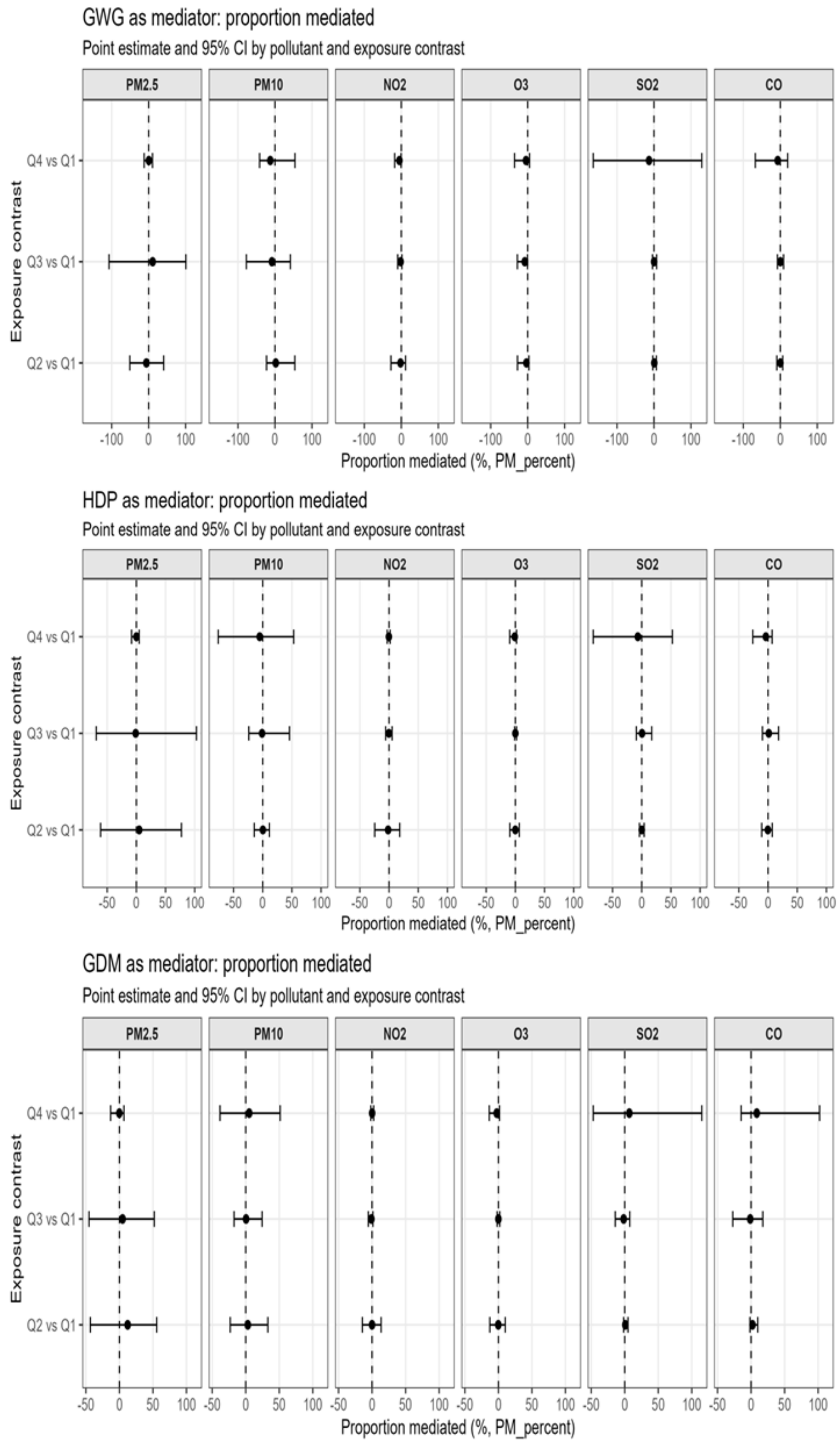

Supplementary Figure S8. The proportion mediated values of gestational weight gain, gestational hypertensive disorders, and gestational diabetes on the relationship between air pollution and oligohydramnios-mediation analyses. GWG: gestational weight gain, HDP: gestational hypertensive disorders, GDM: gestational diabetes.

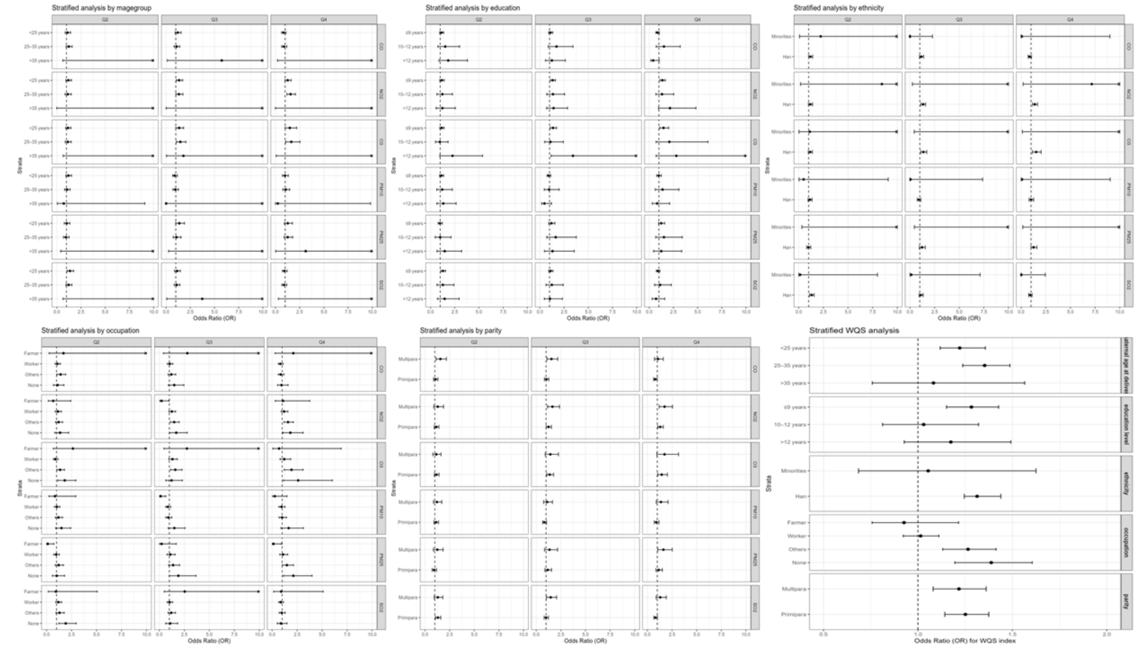

Supplementary Figure S9. Comparison of the effects of air pollution and oligohydramnios among different subgroups. (A) ~ (E): Single pollutant stratification analysis, and ORs represent the contrast between quartiles of each pollutant (Q2–Q4 vs. Q1 as the reference). (F): Mixed pollutant stratification analysis. and ORs reflects the change in oligohydramnios risk associated with a one-unit increase in the WQS index.

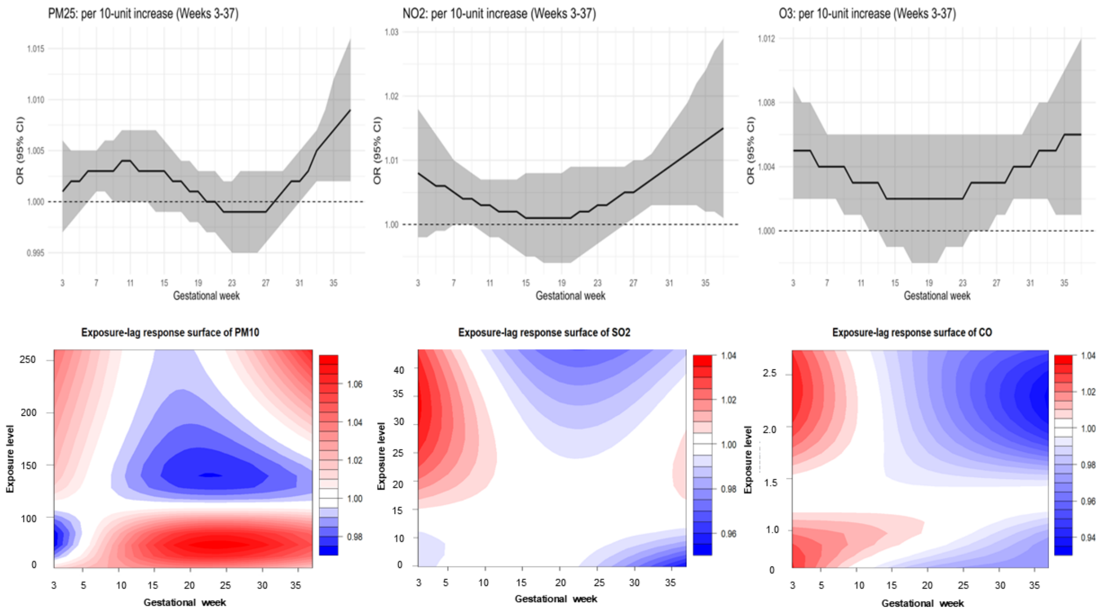

Supplementary Figure S10. The weekly impact of a single pollutant on oligohydramnios calculated by DLNM - pollutant as a continuous variable. DLNM: Distributed lag non-linear model. (A) ~(C): The association between pollutants and oligohydramnios shows a linear effect - the OR and 95% CI for different gestational weeks for every 10 units increase in pollutants. (D)~(F): The association between pollutants and oligohydramnios shows a non-linear effect - the OR and 95% CI for different gestational weeks at different pollutant concentrations.
